# A global mandate to strengthen emergency, critical and operative care

**DOI:** 10.2471/BLT.23.289916

**Published:** 2023-04-01

**Authors:** Lia Tadesse, Noor Hisham Abdullah, Heitham Mohamed Ibrahim Awadalla, Scott D’Amours, Ffion Davies, Niranjan Kissoon, Wayne Morriss, Bisola Onajin-Obembe, Teri Reynolds

**Affiliations:** a Ministry of Health, Addis Ababa, Ethiopia.; b Ministry of Health, Putrajaya, Malaysia.; c Federal Ministry of Health, Khartoum, Sudan.; d International Association for Trauma Surgery and Intensive Care, Zurich, Switzerland.; e International Federation for Emergency Medicine, Victoria, Australia.; f Global Sepsis Alliance, Berlin, Germany.; g World Federation of Societies of Anaesthesiologists, London, England.; h The Global Alliance for Surgical, Obstetric, Trauma and Anaesthesia Care (G4 Alliance), New York, United States of America.; i Integrated Health Services, World Health Organization, Avenue Appia 20, 1211 Geneva 27, Switzerland.

The recent decision at the World Health Organization (WHO) Executive Board (EB152/3)^1^ to recommend that the World Health Assembly adopt a resolution on “Integrated emergency, critical and operative care for universal health coverage and protection from health emergencies” provides an extraordinary opportunity for coordinated action in countries. The decision also offers a solid foundation for ongoing advocacy across the global emergency, critical, surgical and anaesthesia care communities. Support among Member States is broad, and the proposed resolution is already formally co-sponsored by over 80 countries spanning all regions.

Emergency, critical and operative care services are an integral part of a comprehensive primary health-care approach and are essential to meet people’s health needs across the life course. Everyone needs primary care every day – whether attention to healthy diet and exercise, coordinated prevention or a chronic treatment regimen. However, most people will also need emergency, critical and operative services during their lifetime, whether for treatment of a laceration, a fracture or a fever late at night; ambulance transport, oxygen or intubation for pneumonia; placement of a chest drain after a road crash; or surgery and anaesthesia for a hernia, obstructed labour or cancer.

Emergency, critical and operative care represent a people-centred continuum, and the delivery of these services is not associated with any single health worker cadre, specialty, condition or setting. For example, nurses deliver as much emergency care around the world as doctors, and both may be trained as specialists of the first hours of care; operative care is understood to span both anaesthesia and surgery services in theatres and ambulatory centres, while surgeons and anaesthesia professionals may deliver emergency, critical and operative care in the course of a day as part of comprehensive peri-operative care and beyond. Critical care may be delivered in an ambulance or in an emergency unit, an intensive care unit or a theatre by technicians, nurses or specialist doctors. Traumatology in some countries is an orthopaedic surgical specialty; in others, a trauma fellowship may focus on critical care for the injured. Only a coordinated and strategic approach to the entire emergency, critical and operative care continuum, including rehabilitative and palliative services, can deliver effective care for people with key high-burden acute conditions, such as injury or sepsis. The distinction between emergency and critical care, between emergency and operative care or between critical and operative care is highly variable by context, and these services are highly interdependent in all contexts.

The recent pandemic revealed pervasive gaps in capacity, and has changed our understanding of health systems and emergency response. While gaps in primary, emergency or critical care directly hindered an effective response, the most disrupted category of health services was elective surgery,^2^ and many countries will struggle with the resulting surgical backlog for decades to come. As major conflicts and devastating earthquakes have overlapped the waning of the pandemic, the need for an integrated approach to emergency, critical and operative care has become even more evident. Millions of people around the world live in a protracted emergency context, and no emergency waits for the previous one to end. Only an integrated approach to the full emergency, critical and operative care continuum, linked effectively to robust primary care, can ensure effective preparedness, readiness and response.

The proposed resolution builds on, integrates and renews the mandate of prior resolutions: WHA56.24 on violence and health;^3^ WHA57.10^4^ on road safety and health;^4^ WHA60.22 on emergency care systems;^5^ WHA64.10 on strengthening health emergency and disaster management capacities and resilience;^6^ WHA68.15 on strengthening emergency and essential surgical care and anaesthesia;^7^ WHA69.1 on strengthening essential public health functions;^8^ WHA70.7 on sepsis;^9^ WHA72.6 on patient safety;^10^ WHA72.16 on emergency care systems for universal health coverage;^11^ and WHA74.7 on preparedness for and response to health emergencies.^12^ We have greatly increased awareness through the mandates of these resolutions and under the banner of global emergency care, global critical care, and global surgery and anaesthesia movements. The proposed resolution amplifies these agendas, providing an integrated and solid foundation for continued action and effective implementation.

Successful implementation of the mandates outlined will require broad engagement of governments, communities, partners and a range of other stakeholders. The authors of this editorial represent the highest levels of government leadership as well as WHO and major global partner organizations; we call on our communities to come together to meet this challenge.
